# Erroneous data: The Achilles' heel of AI and personalized medicine

**DOI:** 10.3389/fdgth.2022.862095

**Published:** 2022-07-22

**Authors:** Thomas Birk Kristiansen, Kent Kristensen, Jakob Uffelmann, Ivan Brandslund

**Affiliations:** ^1^Ishøjcentrets Læger, Ishøj, Denmark; ^2^Institute of Law, University of Southern Denmark, Odense, Denmark; ^3^Public Danish E-Health Portal (Sundhed.dk), Copenhagen, Denmark; ^4^Sundhed.dk International Foundation, Copenhagen, Denmark; ^5^Department of Medical Science and Artificial Intelligence, Institute of Regional Health Research, University Hospital of Southern Denmark Sygehus Lillebælt (SLB), University of Southern Denmark, Odense, Denmark

**Keywords:** AI, artificial intelligence, data quality, personalized medicine, machine learning (ML), deep learning

## Abstract

This paper reviews dilemmas and implications of erroneous data for clinical implementation of AI. It is well-known that if erroneous and biased data are used to train AI, there is a risk of systematic error. However, even perfectly trained AI applications can produce faulty outputs if fed with erroneous inputs. To counter such problems, we suggest 3 steps: (1) AI should focus on data of the highest quality, in essence paraclinical data and digital images, (2) patients should be granted simple access to the input data that feed the AI, and granted a right to request changes to erroneous data, and (3) automated high-throughput methods for error-correction should be implemented in domains with faulty data when possible. Also, we conclude that erroneous data is a reality even for highly reputable Danish data sources, and thus, legal framework for the correction of errors is universally needed.

## Introduction

Artificial intelligence (AI) in healthcare may carry great promise, though in many cases strong clinical evidence for its positive effects is lacking (1). Currently, AI is being developed for many purposes such as automated diagnosing in clinical laboratory medicine (2), for radiological imaging description (3), for monitoring of patients (4), and to stratify the acuity of incoming patients (5).

An ever-increasing availability of digital health data has, alongside computer and mathematical development, been a major driver toward clinical AI. In Denmark, the evolvement of electronic health records (EHR) over more than 20 years has led to an abundance of healthcare data. Also, Denmark has rigorously and systematically gathered registries on its citizens longer than most other country, and although abundant registries also exist in other Nordic countries, Denmark is by some considered a benchmark for high quality registries touching on almost every aspect of life (6). Even so, reputable databases in Denmark face a problem of high-quality data being mixed with poor-quality data. This is problematic since AI depend on the quality of data (7). An example is “IBM Watson Oncology,” a digital physician assistant based on machine learning, that despite a hyped-up and expensive investment, continues to make incorrect recommendations for cancer treatment due to, amongst other issues, problems with mixed data quality (8, 9).

This paper reviews the dilemmas and implications of erroneous data in clinical AI, particularly for use in personal medicine. The remainder of the paper structures as follows: Section 2 describes how AI is trained, section 3 explains how the clinical application of AI is affected by erroneous EHRs section 4 shows how reputable Danish healthcare data are often flawed, section 5 presents the legal regulation of data quality for AI in Denmark and EU, and section 6 discusses how to counter erroneous and flawed health data to progress with personalized medicine.

## AI: Training, validating, and testing

In AI computers can be trained to make decisions and predictions based on past outcomes often relying on *big data*. In other contexts, this is called profiling, but in the context of healthcare this is often referred to as precision medicine or personalized medicine. Much progress in the field of AI and healthcare is done using machine learning (ML) and deep learning (DL). The development and training of AI applications (including ML and DL) involve at least three data sets: a *training set*, a *validation set*, and a *test set* (10). The *training set* is used for building the initial AI model, *the validation* set is used to qualify the performance of the model, and the *test set* is used to qualify the accuracy of the final model.

Data can often be categorized and labeled according to a certain attribute. An AI algorithm can subsequently, be trained to recognize patterns that match this label. This is called supervised training, though, in some cases, it may also be possible to successfully train pattern recognition on training data completely without labels, which is called unsupervised training (11).

In image recognition, for example, algorithms are often trained with images that identify the patterns specific to a certain diagnosis, so that subsequently, the application can recognize the diagnosis by checking for the labeled pattern. This may cause obvious problems in supervised training, because without accurate and optimal classification, the AI application can be trained to recognize imprecise patterns, especially if done by human assessment (12). This can even become self-perpetuating because the imprecisions and systematic errors of an AI application, can subsequently impact the training data for the future (13, 14).

Problems concerning biased and imprecise data for the training of AI have been exhaustively reviewed and discussed in the literature (15–19). Still, large amounts of training data with stochastic errors can train an AI application which is robust *on average*, and which *on average* performs well.

## Clinical application of AI and the problem of erroneous EHRs

Once an AI application is trained, validated, and tested, some AI applications will become clinically implemented. Here the flow of data is reversed. The application is now fed with specific data, often concerning individual patients. These data are referred to as *input data*. When an AI application is fed with *input data*, the application can come up with statistical predictions, so-called *output data*.

An often-overlooked problem stems from stochastic errors in the recorded input data retrieved from real life EHRs. If there are significant errors in the input data from EHRs, these erroneous input data can lead to erroneous outputs. Even perfectly trained AI applications can produce faulty outputs if fed with erroneous inputs. Thus, some patients will experience that that AI decision support systematically leads to wrong decisions. This can often be attributed to erroneous input data. No matter how perfect AI performs on average, it will often make faulty predictions if fed with incorrect input data.

For example, a faulty diagnosis concerning one patient, can result in incorrectly estimated treatment decisions when this diagnosis (of a condition which the concerned patient is not suffering from), is fed to an AI application. Without correction of such an error, this patient may therefore systematically receive improper treatment when using AI. This is discrimination by erroneous data.

Data in EHRs are generally recorded by physicians in an unstructured manner during or after patient examination. This process is intrinsically subject to great uncertainty due to differences in interpretations and assessments of the physicians. Thus, many recorded findings and diagnoses are by nature uncertain. Also, it is important to stress that data collection in an everyday setting is not easy.

Paraclinical data and patient imagery are by nature most accurate, and in some cases the amount of data concerning one patient (e.g., full genomes and MRI-scans) are so plentiful they can in themselves be considered *big data*, thus making them robust to stochastic errors. However, the accuracy of paraclinical data depends on differing devices from different manufacturers and models, often leading to data that are not directly comparable (20).

Also, much technical equipment changes precision over time and needs to be calibrated regularly. Therefore, if paraclinical data and imagery from an entire healthcare sector is to be used for AI and personalized medicine, this sector may need to be synchronized in terms of data collection, equipment uniformity and apparatus calibration. This is not an easy task and could prove expensive. In addition, altering the focus of data collection to comply with the needs of AI might be detrimental for patient care. Delivering high quality patient care should always be primary focus and the collection of high-quality data secondary. Also, emphasis on uniformity may lead to centralization, and may even slow local progress and innovation at hospital level, though with strict focus on standardization, this is not an impossible task.

As will be shown in section 4, even highly regarded Danish registries contain erroneous and flawed data. Since even these benchmark data are flawed, it is likely that mixed data quality is universal across countries and could thus pose an Achilles' heel of AI and personalized medicine.

## The danish case: Is excellence in data quality good enough for AI?

Danish healthcare registries, as well as civil registries, are by some considered among the best and most complete (6). The Danish government and national healthcare system have through half a century registered the entire population, thus providing access to detailed patient data, facilitating epidemiological and pharmaceutical research. In this context, Denmark as a nation can in some respects be considered one complete cohort (21) which may be ideal for the development of personalized medicine (22).

The building of vast databases in Denmark has been facilitated using the Danish Civil Registration System (CRS). The CRS is an administrative registry, which as of 1972 contains individual-level information on all persons residing in Denmark and Greenland. By January 2014, the CRS had cumulatively registered 9.5 million individuals and more than 400 million person-years of follow-up (23). A unique ten-digit Civil Personal Register number assigned to all persons in the CRS allows for unambiguous individual-level record linkage of Danish registers, enabling more than 160 public health databases to be continuously feed directly from EHR.

**Figure 1:**
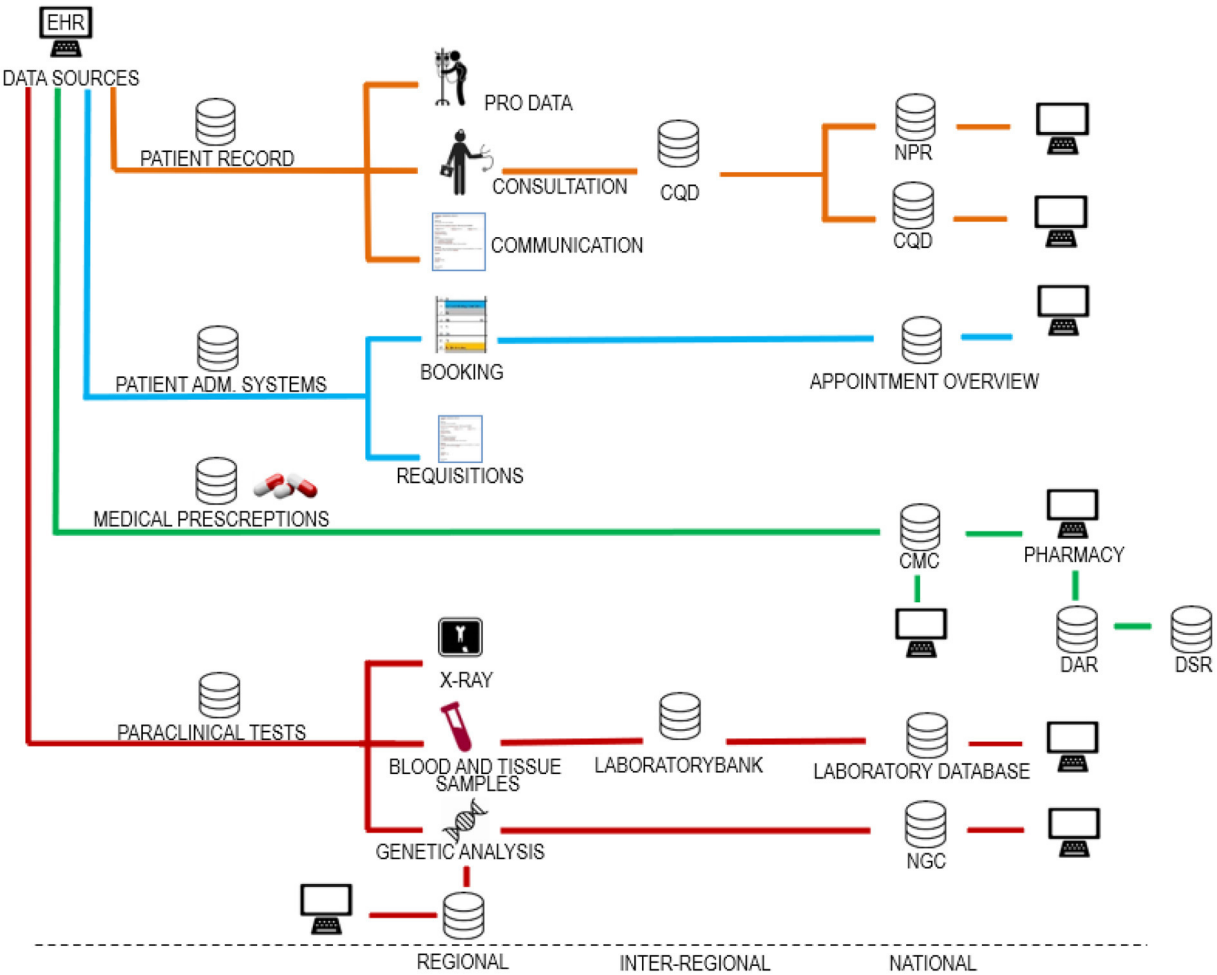
Danish digital infrastructure for health data. The figure shows different data categories divided into regional, interregional and national systems. Source systems making up the digital infrastructure can be categorized in relation to four data categories. (1) data collected for use in the patient record, (2) data collected for administration of the patient course, (3) data on drugs for use in the administration of drugs, and (4) paraclinical data. As shown, it is a basic principle that the same health data are usually stored both regionally, nationally, and in some cases also inter-regionally. In this way, data control is divided between the regions and the state. This data redundancy may be deliberate but may also be by chance as the infrastructure has grown organically over many years. The figure is included in a textbook by Kristensen (24). EHR, electronic health record; PRO data, patient recorded outcome; CQD, clinical quality databases; NPR, national patient register; PAS, patient administrative systems; CMC, common medicine card; DAR, drug administration register; DSR, drug statistics register; NGC, national genome center.

Danish EHRs are based on distributed databases connected in a digital infrastructure where healthcare operators can share and access patient data across organizational boundaries. A simplified map of the Danish digital infrastructure for healthcare data is presented in Figure 1. The development of the digital infrastructure has been ongoing for more than a quarter of a century based on a combination of statutory reporting obligations and data processing agreements between the healthcare operators. The evolvement of this digital infrastructure has not been systematically planned; thus, it is not easy to make an overall overview.

Within the infrastructure, data will inherently be of varying quality. This is illustrated by an analysis of data quality from 2019, which found that data on Danish medical prescriptions contain numerous and frequent incorrect registrations (25). Likewise, 12% of the patients in the Danish registry of diabetes did not have diabetes (26, 27) and in a Danish registry on congenital heart disease 36% of diagnoses where misclassified (28). This pertains not only to Denmark. In one study including patients from 20 countries, 62% of those registered as having chronic obstructive pulmonary disease, did in fact not have the disease (29).

Data sharing means that errors are automatically transferred within the digital infrastructure (Figure 1). As IT-systems are often developed over time, data will often be added, removed, and reclassified. This process may result in data gaps and incorrect registrations. A further obstacle for AI is the fact that errors in Danish health records are never directly corrected. Instead, corrections, if done, are made through later additional notes. Therefore, erroneous data remain and accumulate in the record, thus obscuring AI.

The accumulation of false diagnoses and findings should cause great concern when combined with AI. Clinical decision support tools fed with accumulated false diagnoses and findings may lead to serious overtreatment of patients and thus reverse the promise of AI as beneficial to health economics. Thus, the opposite may very well-come true (30).

## Legislation for the use of health data for AI

In Denmark AI intended for medical purposes is regulated by EU law. Medical devices are in EU law defined as any instrument, apparatus, appliance, software, implant, reagent, material, or other article intended by the manufacturer to be used, alone or in combination, for human beings for medical purposes (31, 32). Medical devices thus include both stand-alone AI applications that work independently of hardware and AI applications that are integrated within a medical device.

Medical purposes are broadly formulated in the regulations, leaving it up to Member States to decide whether a software should be considered a medical device (33). However, only applications intended for medical purposes are to be considered (34). Furthermore, the medical device should include functions that goes beyond storing, displaying, and sharing health data, which means a computer is not a medical device. For example, diagnostic applications used in image processing software to scan images and data from multiple patients are considered medical devices. By contrast, applications that, on the basis, of information on drug prescriptions, search for information on side effects in scientific literature and databases are not considered medical devices.

It is the responsibility of the Member States on a case-by-case basis to assess whether a given AI application should be considered a medical device. The regulations give Member States the authority to lay down safety, quality, and performance requirements in national legislation for products without a medical purpose, if they have characteristics and risk profiles that are like that of medical devices (31). Applications that strictly do not fall within the definition of medical devices, may therefore none the less be categorized as medical devices in national legislation.

A *Conformité Européenne* (CE) mark on a product indicates that the manufacturer or importer comply with relevant EU legislation, and only medical devices meeting the requirements for a CE marking can be marketed. Requirements for approval vary with the risks associated with the devices in question. The higher the risk class, the stricter the safety requirements. The Regulation on Medical Devices operates with four risk classes designated by I, IIa, IIb, and III (31). Class I is associated with the lowest risk, while class III is associated with the highest risk. In this classification, which is new, virtually all software is classified as class IIa, there are however several exceptions to this. The manufacturer is solely responsible for this risk classification. The approval is based on review of the documentation of the software in question, and clinical test on humans is only required for class III devices and under certain circumstances only. There are no EU requirements for the quality of the health data used for development of medical AI applications.

Thus, the legal framework surrounding AI, and in particular AI for healthcare, does not set specific requirements to the quality of data used for training and input in AI.

## Conclusion: Toward minimizing discrimination by erroneous data

As outlined, erroneous healthcare data may pose dire consequences for AI and therefore for patients.

Therefore, mechanisms should be incorporated that protect patients' rights and reduce the risk of improper treatment of specific patients when using AI. This may to some extend be managed by making requirement for human refereeing of AI applications.

A requirement for human refereeing ensures that many faulty decisions can often be ruled out by human assessment. However, this is not sufficient, since the use of automated decision support, may lead to overreliance on the support-system and subsequently to expert deskilling (35, 36). Other actions such as regulating quality of input data for AI and/or formally requiring randomized clinical trials of AI before clinical application could lessen the dependence on human refereeing.

When developing AI and personalized medicine, a focus on data with the highest quality, in essence paraclinical data and digital images, will probably lead to quickest advances. Furthermore, there is a need for relevant legislation to provide a better opportunity to correct source data when used for AI. Also, automated high-throughput methods for error-correction (37) are much needed in domains with faulty data if AI is to be implemented with success. However, it is likely that such automated processes can only partially correct errors and may in fact themselves occasionally cause errors. Thus, in many cases, the patients themselves would be the ones in the best position to find errors, and with large data sets, the patients will often be the only ones who have the resources to find errors. Consequently, patients should be granted simple access to the input data that feed the AI application. As all patients will not correct errors and many errors are not obvious to layman, many complementary strategies to correct erroneous data are needed.

Furthermore, the legal foundation for correction of erroneous data is needed. Thus, in Denmark parliament has begun legal work on how to correct serious errors in patient records (38), and in May 2022 an EU Proposal for a Regulation on the European Health Data Space (39) was presented introducing a right for patients to request changes to erroneous data online (39, 40).

As we have shown Danish health registries are often flawed, and in this context, we suggest that much real-world clinical data are erroneous by nature, or at least only correct to the best knowledge of the physicians. The errors may indeed be so plentiful that it would never be possible to correct them all. It could also be that training of an algorithm on perfectly curated data may make it unfit for the real world.

In conclusion, if the issues of erroneous health data are not properly addressed erroneous data could indeed be an Achilles' heel of clinically applied AI. To counter such problems, we suggest 3 steps: (1) AI should focus on data of the highest quality, in essence paraclinical data and digital images, (2) patients should be granted simple access to the input data that feed the AI, and granted a right to request changes to erroneous data, and (3) automated high-throughput methods for error-correction should be implemented in domains with faulty data when possible.

## Author contributions

TK: conceptualization, writing—original draft (Introduction, AI: Training, validating, and testing, Clinical application of AI and the problem of erroneous EHRs, The Danish case: Is excellence in data quality good enough for AI?, Conclusion: Toward minimizing discrimination by erroneous data), visualization—reviewing and editing (Figure 1), KK: conceptualization, writing—original draft (Legislation for the use of health data for AI), visualization—original conceptualization and original draft (Figure 1), and writing—review and editing. JU: visualization—reviewing and editing (Figure 1), and writing—review and editing. IB: conceptualization and writing—review and editing. All authors contributed to the article and approved the submitted version.

## Conflict of interest

The authors declare that the research was conducted in the absence of any commercial or financial relationships that could be construed as a potential conflict of interest.

## Publisher's note

All claims expressed in this article are solely those of the authors and do not necessarily represent those of their affiliated organizations, or those of the publisher, the editors and the reviewers. Any product that may be evaluated in this article, or claim that may be made by its manufacturer, is not guaranteed or endorsed by the publisher.
